# Spontaneous retroperitoneal haematoma causing acute haemodynamic collapse: a case report

**DOI:** 10.1093/jscr/rjag088

**Published:** 2026-02-27

**Authors:** Sandeepa Dadigamuwage, Vimarshini Samarakoon, Rajesh Thengungal Kochupapy

**Affiliations:** Colorectal Surgery Department, University Hospitals Plymouth NHS Trust, Derriford Road, Crownhill, Plymouth, Devon, PL6 8DH, United Kingdom; Torrington Cardiac Intensive Care Unit, University Hospitals Plymouth NHS Trust, Derriford Road, Crownhill, Plymouth, Devon, PL6 8DH, United Kingdom; Colorectal Surgery Department, University Hospitals Plymouth NHS Trust, Derriford Road, Crownhill, Plymouth, Devon, PL6 8DH, United Kingdom

**Keywords:** retroperitoneal haematoma, spontaneous haemorrhage, computed tomography, CT angiography, shock, blood transfusion refusal

## Abstract

Spontaneous retroperitoneal haemorrhage is a rare but potentially life-threatening cause of acute abdominal pain and shock. We report a 70-year-old man with polycythaemia on aspirin who presented with sudden right-sided abdominal pain, groin discomfort, leg paraesthesia and haemodynamic instability. Computed tomography (CT) demonstrated a large right-sided retroperitoneal haematoma with active arterial extravasation, confirmed on CT angiography. Management was complicated by the patient’s refusal of blood transfusion despite counselling. Although endovascular embolisation was planned, his clinical deterioration coincided with interventional radiology being unavailable due to another emergency. He progressed to refractory shock and died 21 hours after presentation. This case highlights the need for early CT imaging, timely access to embolization and sensitive communication when patient beliefs limit treatment options in acute haemorrhagic emergencies.

## Introduction

Spontaneous retroperitoneal haematoma is a rare but potentially life-threatening condition occurring without trauma or iatrogenic injury. It accounts for around 1% of acute abdominal emergencies and carries a mortality rate of 20%–30%, largely due to delayed recognition and sudden haemodynamic collapse [1, 2]. Spontaneous retroperitoneal haematoma is most often associated with anticoagulation, advanced age, vascular fragility or underlying comorbidities, although idiopathic cases are well described [3].

Clinical presentation is variable, ranging from flank or abdominal pain to profound shock, and symptoms may be non-specific until significant bleeding has occurred. Contrast-enhanced computed tomography (CT) is essential for diagnosis, allowing confirmation of bleeding, assessment of extent and detection of active extravasation, a key predictor of deterioration and need for intervention [4].

Management is guided by haemodynamic status. Stable patients may be managed conservatively, while active bleeding or instability usually requires urgent endovascular embolization or surgery [5].

## Case report

A 70-year-old man presented with sudden onset right lower abdominal pain radiating to the groin, accompanied by numbness of the right leg. The pain began while rising from a sunbed. His medical history included polycythaemia managed with daily aspirin, and he was under regular haematology follow-up. He reported minor trauma to the right flank two weeks prior.

On examination, he appeared pale and distressed. His observations showed hypotension with a systolic blood pressure of 90 mmHg and tachycardia. Abdominal examination revealed marked right lower abdominal tenderness, and neurological assessment identified paraesthesia of the right leg up to the knee. Howship–Romberg sign was positive. Based on the unilateral groin pain and neurological findings, an obturator hernia was initially suspected.

Initial laboratory results showed a haemoglobin of 150 g/L and raised inflammatory markers. A summary of investigations on admission and at 13 hours is provided in Table 1.

**Table 1:** Summary of laboratory investigations at admission and 13 hours post-presentation.

Parameter	On admission	13 Hours after admission	Reference range
Haemoglobin (g/L)	150	112	130–175 g/L
White Cell Count (×10^9^/L)	53.6	84.9	3.6–9.2 × 10^9^/L
Neutrophils (×10^9^/L)	46.4	71.9	1.7–6.2 × 10^9^/L
Platelets (×10^9^/L)	567	814	150–450 × 10^9^/L
CRP (mg/L)	12	27.1	0.1–5 mg/L
Urea (mmol/L)	8.7	11.7	2.5–7.8 mmol/L
Creatinine (μmol/L)	94	136	64–104 μmol/L
Sodium (mmol/L)	138	137	133–146 mmol/L
Potassium (mmol/L)	5.4	4.9	3.5–5.3 mmol/L
Lactate (mmol/L)	3.2	14.0	0.5–2.0 mmol/L
pH	7.39	7.14	7.35–7.45
PaO₂ (kPa)	9.2	3.37	10–13 kPa
PaCO₂ (kPa)	5.3	5.52	4.7–6.0 kPa
SaO₂ (%)	93.1	23.0	95–100%
Bicarbonate (mmol/L)	N/A	13.5	22–26 mmol/L

Abbreviations: CRP, C-reactive protein; pH, Potential of hydrogen; PaO₂, Partial pressure of arterial oxygen, PaCO₂, Partial pressure of arterial carbon dioxide; SaO₂, Arterial oxygen saturation.

**Figure 1:**
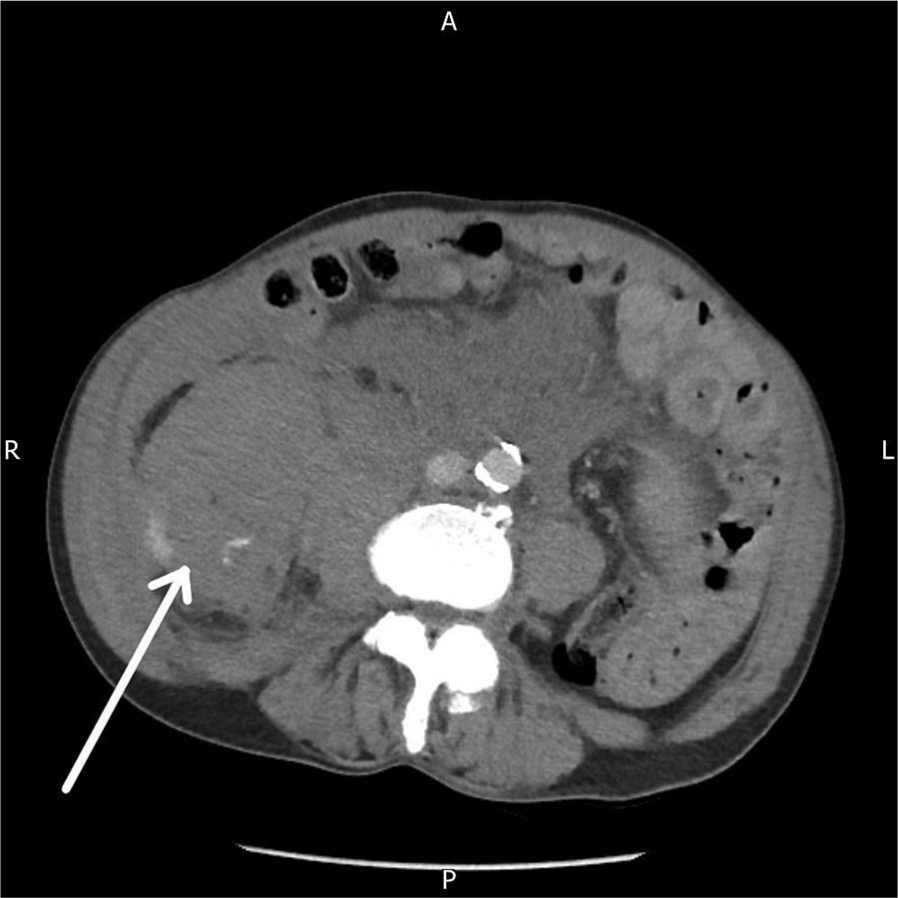
Axial contrast-enhanced CT abdomen and pelvis demonstrating a right-sided retroperitoneal haematoma with evidence of active contrast extravasation.

A contrast-enhanced CT abdomen and pelvis performed five hours after presentation demonstrated a large right-sided retroperitoneal haematoma extending from the perinephric region into the psoas compartment, with clear evidence of active arterial extravasation (Fig. 1). Splenic enlargement with patchy enhancement was also noted, felt to represent a perfusion abnormality. No underlying mass or vascular lesion was identified.

Given the initial haemodynamic improvement following fluid resuscitation, and after discussion with interventional radiology, embolization was deferred, with a plan for close monitoring. Thirteen hours after admission, his haemoglobin had fallen significantly, prompting a CT angiogram. At that point, the patient disclosed an advance decision refusing any blood or blood-product transfusion due to concerns about mRNA vaccine contamination in donor blood. His decision-making capacity was formally assessed and documented.

**Figure 2:**
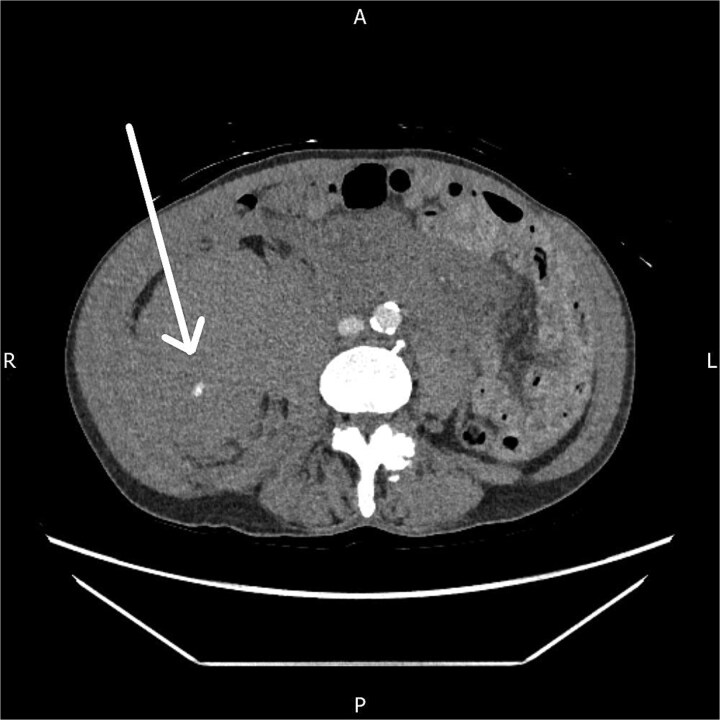
Axial CT angiogram showing persistent right-sided retroperitoneal haematoma with ongoing active contrast extravasation.

The CT angiogram, performed 14 hours after presentation, showed a further increase in the size of the haematoma with ongoing active arterial contrast extravasation (Fig. 2). Despite the clear need for embolization, the interventional radiology team was unavailable due to a concurrent emergency life-saving procedure.

The patient subsequently deteriorated rapidly with worsening hypotension and respiratory failure. Emergency laparotomy was considered but deemed unsafe without transfusion support. Despite maximal non-blood resuscitation and intravenous tranexamic acid, he suffered a cardiac arrest and died approximately 21 hours after admission.

## Discussion

Spontaneous retroperitoneal haematoma is an uncommon but serious condition that typically occurs in anticoagulated or medically complex patients, although minor unnoticed trauma may be contributory [6]. The retroperitoneal cavity can accommodate large volumes of blood before producing overt clinical signs, accounting for the delayed or subtle presentation seen in many cases. Patients may initially report flank or abdominal pain, but rapid progression to haemodynamic instability is well recognized [7].

CT is central to diagnosis, confirming the presence and extent of bleeding and identifying features such as active contrast extravasation, which strongly predict deterioration and the need for intervention [8]. Haemodynamically stable patients without radiological evidence of ongoing haemorrhage may be managed conservatively with close monitoring, whereas worsening instability or active bleeding usually warrants urgent endovascular embolization, now considered the preferred minimally invasive approach [9]. Surgical exploration is reserved for refractory cases or when interventional radiology is unavailable [10].

In this case, initial stabilization was followed by sudden deterioration, and although embolization was planned, access to interventional radiology was delayed due to a concurrent emergency. This reflects a practical challenge: the window for intervention in spontaneous retroperitoneal haematoma can be narrow, and system pressures may critically influence outcome. Additionally, management was complicated by the patient’s refusal of blood transfusion due to concerns about vaccine-related contamination. Such beliefs are increasingly encountered and require clear, empathetic communication, yet may limit life-saving options at crucial moments [11].

This case underscores the rapid clinical trajectory of spontaneous retroperitoneal haematoma and the importance of timely imaging, coordinated escalation and sensitive, patient-centred decision-making.

## References

[1] Sunga KL, Bellolio MF, Gilmore RM et al. Spontaneous retroperitoneal hematoma: etiology, characteristics, management, and outcome. J Emerg Med 2012;43:e157–61. 10.1016/j.jemermed.2011.06.006 21911282

[2] Chan YC, Morales JP, Reidy JF et al. Management of spontaneous and iatrogenic retroperitoneal haemorrhage: conservative management, endovascular intervention or open surgery? Int J Clin Pract 2008;62:1604–13. 17949429 10.1111/j.1742-1241.2007.01494.x

[3] Wytock DH, Lebron RP, Milano CA et al. Spontaneous retroperitoneal hemorrhage: management in the endovascular era. J Vasc Surg 2019;70:573–9.

[4] Takahashi M, Yoshida K, Shimura T et al. Spontaneous retroperitoneal hematoma: role of multidetector CT in diagnosis and management. Clin Radiol 2010;65:751–7.

[5] García-Moncó JC, Antuña S, Escribano D et al. Management of spontaneous retroperitoneal hematoma: a multicenter experience. Eur J Trauma Emerg Surg 2019;45:1107–14.30167738

[6] Sunga KL, Bellolio MF, Gilmore RM et al. Spontaneous retroperitoneal hemorrhage: diagnosis and management. Int J Emerg Med 2012;5:51. 10.1016/j.jemermed.2011.06.006 21911282

[7] Chan YC, Morales JP, Reidy JF et al. Endovascular management of spontaneous retroperitoneal haemorrhage: indications and outcomes. Int J Clin Pract 2008;62:1604–13. 17949429 10.1111/j.1742-1241.2007.01494.x

[8] Takahashi M, Yoshida K, Shimura T et al. Multidetector CT in spontaneous retroperitoneal hematoma: predicting active bleeding. Clin Radiol 2010;65:751–7.

[9] Barral M, Pommier R, Vidal V et al. Endovascular management of spontaneous retroperitoneal hematoma: safety and outcomes. Eur J Radiol 2019;112:62–8.

[10] D'Amelio LF, Pryor RW, Elliott DW. Retroperitoneal hemorrhage: diagnosis and treatment. Ann Surg 1982;195:270–5.7059239

[11] Forkin KT, Wickham K, Pasupathy S et al. Patient concerns regarding blood transfusion and vaccine-related misinformation: implications for communication. Anesthesiology 2024;140:456–64.

